# Rapport in same and mixed neurotype groups of autistic and non-autistic adults

**DOI:** 10.1177/13623613251320444

**Published:** 2025-02-24

**Authors:** Sarah J Foster, Robert A Ackerman, Charlotte EH Wilks, Michelle Dodd, Rachel Calderon, Danielle Ropar, Sue Fletcher-Watson, Catherine J Crompton, Noah J Sasson

**Affiliations:** 1 The University of Texas at Dallas, USA; 2 University of Nottingham, UK; 3 The University of Edinburgh, UK

**Keywords:** autism, double empathy, group interaction, neurotype, rapport

## Abstract

Although autistic adults may establish better dyadic rapport with autistic relative to non-autistic partners, it is unclear whether this extends to group settings. The current study examined whether rapport differs between autistic groups, non-autistic groups, and mixed groups of autistic and non-autistic adults, and whether differing diagnostically from the rest of the group results in lower rapport. One hundred and forty-three adults were assigned to one of four group types of four participants each: all-autistic, all-non-autistic, non-autistic majority (three non-autistic, one autistic), and autistic majority (three autistic, one non-autistic). Groups participated in a 5-minute building activity and afterwards completed a 5-item rapport measure assessing their experience. The all-autistic groups did not differ on overall rapport from the all-non-autistic groups and had significantly higher rapport on two items (enjoying the interaction and friendliness of the group) than both types of mixed groups. At the individual level, autistic participants expressed more ease and enjoyment when interacting with other autistic adults relative to non-autistic adults, and their rapport declined as more non-autistic participants were included in the group. In contrast, rapport for non-autistic participants remained relatively consistent regardless of group composition. We discuss potential reasons why autistic participants were more affected by group composition than non-autistic participants.

**Lay abstract:**

Autistic adults sometimes get along better with other autistic people compared to non-autistic people, but so far this has only been studied in two-person interactions. This study examined how well autistic and non-autistic people develop rapport in a group setting and whether rapport differs when group members share or do not share a diagnosis. We assigned 143 adults to 36 groups of four adults each. Some groups only had autistic members, some only had non-autistic members, and some were “mixed” groups of autistic and non-autistic members. Groups participated in a tower-building task for 5 minutes and afterwards completed a survey about rapport with the group. The groups of all-autistic participants expressed that their interactions were more enjoyable and friendly than the mixed groups. Autistic participants reported lower rapport when interacting with non-autistic adults, while non-autistic participants reported similar rapport whether interacting with autistic or non-autistic group members. Overall, findings are not consistent with a social deficit model of autism, as autistic adults often established rapport with partners in a group setting. Their level of rapport, however, depended strongly on the social context, particularly whether other autistic people were also in the group.

Autism is clinically defined by difficulties in social interaction (American Psychiatric Association, 2013). Historically, research investigating the mechanisms of these difficulties has emphasized social cognitive differences within the autistic individual (Sasson et al., 2012), such as theory of mind (Baron-Cohen et al., 1985), face processing (Rutherford et al., 2002; Uljarevic and Hamilton, 2013), and use of eye contact (Senju & Johnson, 2009). Each of these has been targeted for intervention with the implicit assumption that normalizing autistic peoples’ social thinking and behavior would improve their social experiences (Pallathra et al., 2019). However, social skills and social cognitive training programs have largely not translated to real-world social improvements for autistic people (Bottema-Beutel et al., 2018; Fletcher-Watson et al., 2014; Gates et al., 2017; Palmen et al., 2012), in part because social interaction is not an individual skill but rather a dynamic process between at least two people (Bolis & Schilbach, 2018).

An alternative framework for understanding the social interaction difficulties often experienced by autistic people is the double empathy problem (DEP; Milton, 2012). The DEP posits that differences in thinking and communication between autistic and non-autistic people contribute to bi-directional social misunderstandings (Milton, 2012). Thus, although a long history of autism research has established that autistic people often struggle to infer the thoughts, feelings, and beliefs of non-autistic people (Sasson et al., 2012), the DEP emphasizes that the reverse happens too. Non-autistic people misinterpret autistic mental states (Edey et al., 2016; Sheppard et al., 2016), evaluate autistic people negatively, and deny them social opportunities (S. J. Foster et al., 2024; Sasson et al., 2017), express and recognize emotion differently than autistic people (Keating & Cook, 2020), and communicate less effectively with autistic people (Crompton, Hallett, et al., 2020). In contrast, autistic people often experience better communication (Crompton, Hallett, et al., 2020) and establish better rapport (Crompton, Sharp, et al., 2020) with each other compared to non-autistic people.

Rapport is characterized by mutual feelings of positivity, attentiveness, affinity, and harmony within social interactions (Bernieri, 2014; Tickle-Degnen, & Rosenthal, 1990), and it facilitates social connection and the development and maintenance of interpersonal relationships (Cappella, 1990). Relevant to the DEP, rapport is defined interpersonally rather than individually (Bernieri, 2014) and is often more readily established between members of a shared social or identity group relative to out-group members (Miles et al., 2011). Research comparing matched and mixed neurotype dyads of autistic and non-autistic participants indicates that rapport is selectively reduced in mixed interactions, with autistic participants establishing higher rapport with autistic partners (Crompton, Sharp, et al., 2020). Several observable markers or rapport differ for autistic and non-autistic participants, such as a greater role of mutual gaze and verbal backchanneling for non-autistic partners (Rifai et al., 2022). Furthermore, autistic adults tend to disclose more about themselves to other autistic partners and do not demonstrate the non-autistic preference for future interaction with non-autistic partners (Morrison et al., 2020).

However, rapport among and between autistic and non-autistic people to this point has only been studied experimentally in dyads. Social interactions also occur in group settings, whether at work with colleagues, at school with students and teachers, or out in the community with friends and family. Within autistic groups, autistic people often report enhanced social experiences. For example, autistic adolescents gravitate toward interacting with autistic peers in school group settings (Chen et al., 2021), autistic adults report a greater sense of belonging among other autistic people (Crompton, Hallett, et al., 2023), and autistic students in higher education settings describe familiarity and connection with peers as a contributor to positive group work experiences (Drexler, 2024). As a minority, autistic people are most commonly not in autistic spaces but rather need to navigate predominantly non-autistic environments. Many adopt conscious and unconscious strategies to try to fit in and avoid discrimination (Pearson & Rose, 2021). Consistent with the DEP, they may still struggle to establish rapport with non-autistic peers and co-workers (Crompton, Sharp, et al., 2020; Sosnowy et al., 2018, 2019). The current study seeks to examine whether rapport for autistic and non-autistic people differs in group settings when they are in the diagnostic minority compared to the diagnostic majority relative to the rest of the group.

One provocative implication of the DEP is that non-autistic people might be expected to experience something similar within predominantly autistic social spaces. In such situations, non-autistic people would differ from the majority. This could result in difficulty fitting into the group, forging understanding, and establishing rapport. To our knowledge, this has yet to be empirically investigated. Such a result, if found, would reinforce the conjecture that social skill and success are contextually dependent on compatibility between autistic and non-autistic social partners rather than inherent social abilities.

One group environment that may facilitate rapport between and among autistic and non-autistic people is collaborative interaction (Zhang et al., 2020). Collaboration represents a unique social environment where many forms of interactions can be observed; it involves blending insights to develop new ideas, and when done effectively, can result in a better product than what an individual produces alone (Chandler-Olcott & Hinchman, 2019). Collaborative spaces also offer more opportunities for both positive and negative social interactions. People often expect group members to respond to a situation in the same way they would, and shared expectations predict less competitive and more collaborative group behaviors (Ladbury & Hinsz, 2018), suggesting that there may be negative social consequences in these spaces if one does not identify with in-group members (Feather, 1994; Jetten et al., 1997). A collaborative task demand therefore may offer a unique environment to examine rapport between autistic and non-autistic people in group settings.

The current study examined whether rapport differs in autistic groups, non-autistic groups, and mixed groups of autistic and non-autistic adults during a collaborative task. We also probe whether participants’ levels of rapport depend upon their own diagnosis, the diagnostic composition of the other group members, and the degree to which the participants’ diagnosis matches the diagnostic status of others in the group.

We hypothesized that mixed groups of autistic and non-autistic adults would report lower overall rapport relative to the all-autistic and all-non-autistic groups where there is no variation in diagnostic status. Such a finding would support the DEP by demonstrating that autistic groups are as capable of establishing rapport as non-autistic groups, with rapport selectively reduced within mixed group interactions. We also predicted that participants who were the diagnostic minority in the group, whether autistic or non-autistic, would report lower overall rapport than those in the diagnostic majority. Such a finding would suggest that non-autistic people, like autistic people, struggle to establish rapport in spaces that are less conducive to their way of socializing.

## Method

### Participants

One hundred and forty-three participants (77 autistic, 66 non-autistic) completed a group interaction task. Testing occurred at The University of Texas at Dallas (*n* = 52), The University of Nottingham (*n* = 43), and The University of Edinburgh (*n* = 48). This sample size was supported using the Monte Carlo simulation capabilities of the mlmpower package (Keller, 2024) in R to determine the minimum effect-size values for the Group Actor–Partner Interdependence Models (GAPIMs) that we could detect with power = 0.80 and Intraclass Correlation Coefficients (ICCs) for the outcome variables of 0.10 and 0.25. Each analysis used 2,000 simulations and assumed a sample size of 34 groups with four people within each group. Simulations showed that we have adequate power (0.78) to detect small-to-medium effect-size values (i.e. *R*^2^ = 0.03) for the actor and partner effects when the ICC is as low as 0.10 (power = 0.86 when ICC = 0.25). We also have sufficient power (0.83) to detect a medium effect-size value (i.e. *R*^2^ = 0.05) for the actor–partner interaction term when the ICC is 0.25.

Participants were recruited from mailing lists, local charities, social media, university research pools, a study website page, and by word of mouth across the three sites. Participants had to be 18 years or older, native or native-level English speakers, and have normal/corrected normal sight and hearing. Participants were excluded at the screening stage if they had a diagnosis of social anxiety or uncontrolled epilepsy. The autistic sample consisted of those with a formal diagnosis (*n* = 60) and those who self-identified as autistic (*n* = 17). The latter group completed the Ritvo Autism and Asperger Diagnostic Scale (Ritvo et al., 2011) and were only included in the study if they scored 72 or above, which indicates a level of autistic traits above a diagnostic threshold (Andersen et al., 2011). Self-identified autistic participants and those with a formal autism diagnosis did not differ in autistic traits measured with the Ritvo (*p* = 0.376). All participants completed the Ritvo Autism and Asperger’s Diagnostic Scale 14-item Screen (RAADS-14; Eriksson et al., 2013), and non-autistic participants were excluded from participating if their scores indicated high levels of autistic traits (score > 14). Sample demographics can be seen in Table 1. Autistic and non-autistic participants did not significantly differ in age (*p* = 0.323), IQ (*p* = 0.51), or ethnicity (*p* = 0.209), but did on gender (*p* < 0.001), primarily due to more autistic participants identifying as non-binary. Demographics for the four group types can be viewed in Table 2. Autistic and non-autistic minority groups did not significantly differ in instances of the diagnostically different participant also being a different gender (autistic minority = 28.6%, non-autistic minority = 16.7%, *p* = 0.91) or ethnicity (autistic minority = 14.3%, non-autistic minority = 16.7%, *p* = 0.61) compared to the rest of the group.

**Table 1.** Descriptive statistics (mean and standard deviation) and group comparisons for participant demographic variables, IQ, and autistic traits.

	Autistic (*n* = 77)		Non-autistic (*n* = 66)		*p*
	M	SD	M	SD	
Age	28.7	11.7	26.8	10.7	0.32
(WASI-II) IQ	113.9	14.5	109.5	12.1	0.51
(RAADS-14) autistic traits	30.8	8.4	5.5	4.2	<0.001
Gender (%)					<0.001
Male	16.9		24.2		
Female	48.1		71.2		
Non-binary	31.2		3.0		
Non-disclosed	3.8		1.5		
Race (%)					0.21
White	72.7		59.1		
Black	2.6		1.5		
Asian	5.2		12.1		
Hispanic/Latine	2.6		1.5		
Middle Eastern	1.3		0		
Mixed/multiple	14.3		22.7		
Other	1.3		3.0		

**Table 2.** Demographics sorted by group type.

	All-autistic		All-non-autistic		Autistic minority		Non-autistic minority		*p*
	M	SD	M	SD	M	SD	M	SD	
Age	29.4	1.6	27.1	1.8	27.5	2.3	26.2	2.4	0.65
(WASI-II) IQ	112.9	1.8	106.4	2.05	114.5	2.6	114.6	2.7	0.03
Gender (%)									<0.001
Male	19.2		27.5		30.8		0.0		
Female	46.2		70.0		57.7		70.8		
Non-binary	32.7		0.0		7.7		29.2		
Non-disclosed	1.9		2.5		3.8		0.0		
Race (%)									0.71
White	73.1		65.0		55.6		66.7		
Black	1.9		2.5		0.0		4.2		
Asian	5.8		7.5		14.8		8.3		
Hispanic/Latine	3.8		2.5		0.0		0.0		
Middle Eastern	1.9		0.0		0.0		0.0		
Mixed/multiple	11.5		22.5		22.2		20.8		
Other	1.9		0.0		7.4		0.0		

The study was approved by The University of Texas at Dallas Institutional Review Board (IRB), The University of Nottingham’s Psychology Research Ethics Committee, and The University of Edinburgh’s Ethics Committee. All participants provided informed consent prior to participating and were compensated for their time.

### Measures and procedures

Participants were assigned to one of four group types of four participants each: all-autistic (*N* = 13), all-non-autistic (*N* = 10), non-autistic majority (three non-autistic, one autistic, *N* = 7), and autistic majority (three autistic, one non-autistic, *N* = 6), for a total of *N* = 36 groups. One non-autistic participant withdrew before the group interaction began, resulting in three participants in one non-autistic majority group. During recruitment, participants were informed that the purpose of the study was to investigate how autistic and non-autistic people learn from each other. However, to prevent the potential confounding effect of select participants disclosing their diagnostic status during the interaction, group members were informed of the diagnostic composition (e.g. autistic majority) of the group prior to the start of the interaction. The diagnostic status of individuals within the group was not shared.

An experimenter started the task by informing the group that they were leaving to fetch the Jenga building materials, leaving them alone together for 3 minutes. This gave participants a brief chance to introduce themselves to each other before the formal task began. Once the experimenter returned, the groups then completed the Jenga tower-building task for 5 minutes. Participants reported their perceived rapport of the group as a whole after completion of the formal task.

### Jenga task

Groups of four were brought into a room and were provided with the following verbal instructions:

You’re going to be playing a game of Jenga in a group of four people. I just need to go and get the Jenga set. I’ll be back in a couple of minutes, and you can just chat with each other while I’m gone.

After leaving the room for 3 minutes, the researcher returned with a large Jenga set of building blocks (block dimensions: 15 to 16.5 cm × 3 cm × 4.9 to 5.3 cm) and said:

“Your task here as a group is to try to build the tower as high as you can before it falls. I’ll wait outside and I’ll come back after five minutes. If it falls before I come back in, you can have another go.”

The instructions were intentionally ambiguous so participants could interpret the task as a collaborative or competitive endeavor. After 5 minutes of building, participants independently completed a rapport measure assessing their experience with the group.

### Rapport

Rapport was measured with a rating scale used in prior studies of autistic and non-autistic interaction (Crompton, Ropar, et al., 2020; Crompton, Sharp, et al., 2020; Rifai et al., 2022). The scale consists of five rapport items rated from 0 to 100 that are added together to constitute an overall rapport composite score (Crompton, Sharp, et al., 2020). Participants rated how enjoyable, easy, successful, friendly, and awkward they found the interaction. Higher scores indicated a better interactive experience, except “awkward,” which was reverse scored to ensure all items were directionally aligned. The five items had a Cronbach’s alpha of 0.819 in this study, which is very good, and comparable to previous use of this measure (Crompton, Ropar, et al., 2020).

### Community involvement

The lead author is an autistic adult. This experiential expertise informed the research questions, post hoc observational analyses, and finding interpretations. The project team and co-authors consisted of autistic and non-autistic researchers who all contributed to the development, design, interpretation, and writing of this study.

### Analytic strategy

To test our first hypothesis that the single neurotype groups (four autistic participants or four non-autistic participants) would report higher rapport relative to the mixed groups with a neurominority member (one autistic participant with three non-autistic members; one non-autistic participant with three autistic members), we specified Multilevel Models (MLMs) with Restricted Maximum Likelihood Estimation within SPSS v. 29 (IBM Corp., Armonk, NY, USA). These MLMs regressed the composite rapport score and each of the five rapport item scores on group condition (all-autistic, all-non-autistic, non-autistic majority, autistic majority) and included a random intercept to account for the non-independence among group members’ responses. The composite score is reported first, and the item analyses are treated as planned post hoc analyses. Pairwise comparisons between the groups were further explored in cases of a significant omnibus effect of group type on rapport items. This study’s desired sample size, variables, hypotheses, and planned analyses were pre-registered on the Open Science Framework (Foster & Crompton, 2024, osf.io/648h7).

To assess whether rapport differed as a function of a participant’s diagnosis and group composition, we then used MLM with restricted maximum likelihood estimation to specify the group actor–partner interdependence model (Garcia et al., 2015). In this model, “actor” refers to the autistic or non-autistic participant, and “partner” refers to the diagnostic composition of the other group members. Participants’ degree of rapport with the group was predicted as a function of the participants’ diagnosis (effect-coded such that −1 = non-autistic, 1 = autistic), the diagnostic composition of the other group members (effect-coded such that −1 = all-non-autistic, −0.33 = non-autistic majority, 0.33 = autistic majority, and 1 = all-autistic), and the degree of similarity of the participant’s diagnosis to that of the diagnostic composition of the other group members (i.e. the actor–partner interaction term). We also modeled a random group intercept to account for the non-independence in the data. We predicted that participants, regardless of diagnosis, would report lower rapport when they are the minority in the group relative to part of the majority.

## Results

**Figure 1.**
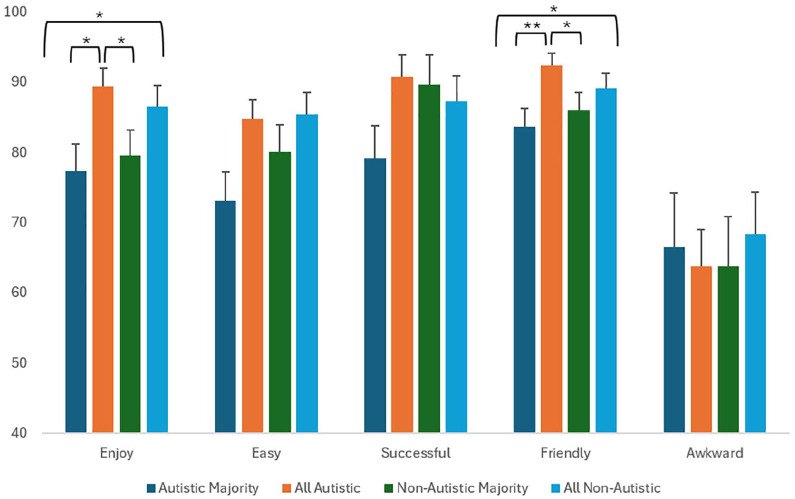
Estimated marginal means (and standard errors) for rapport items for each group type, with 95% confidence intervals. *Denotes a significant difference between groups at *p* < 0.05 and ** at *p* < 0.01. Awkward was reverse scored.

Group-level rapport ratings can be viewed in Figure 1. The all-autistic groups reported the highest mean-level composite rapport and significantly higher ratings for “friendly” and “enjoy” than mixed groups. The MLM analyses revealed that the groups did not significantly differ on composite rapport, but there were significant omnibus effects of group type on group members’ levels of enjoyment, *F*(3, 32.36) = 3.11, *p* = 0.04, and friendliness, *F*(3, 31.18) = 2.99, *p* = 0.046. Follow-up pairwise comparisons showed that the all-autistic groups perceived their group interactions to be significantly more enjoyable and friendlier than both the autistic majority groups (enjoyable, *p* = 0.014; friendly, *p* = 0.010) and the non-autistic majority groups (enjoyable, *p* = 0.034; friendly, *p* = 0.049).

Rapport ratings as a function of the participants’ diagnosis and the group composition can be viewed in Table 3. To test if rapport differed for autistic and non-autistic participants depending on whether they were in the neuromajority or neurominority, we then used MLM with restricted maximum likelihood estimation to specify the group actor–partner interdependence model (Garcia et al., 2015). There was a significant actor effect for participant diagnosis (*b* = −21.28, SE = 8.51, *p* = 0.014). Non-autistic participants reported greater levels of rapport with other group members on average (M = 419.29) compared to autistic participants (M = 376.73), a pattern that seems to be driven by lower ratings of rapport provided by autistic participants toward non-autistic participants. There was also a significant partner effect for the diagnostic composition of the other group members (*b* = 21.19, SE = 10.58, *p* = 0.048), indicating that participants reported greater rapport when more of the group members were autistic (e.g. M = 419.20 when all other group members were autistic) versus non-autistic (e.g. M = 376.83 when all other group members were non-autistic). However, the interaction term was not significant (*p* = 0.145).

There was a significant actor similarity effect for both easy (*b* *=* 5.43, SE = 2.51, *p* *=* 0.033) and enjoy (*b* = 5.89, SE = 2.59, *p* = 0.026). Autistic participants’ rapport ratings of the ease and enjoyment of the interaction decreased as more non-autistic participants were added, while non-autistic participants maintained more similar ratings of easy and enjoy regardless of whether they were in the majority or minority (see Table 3). There was also a significant partner effect of the diagnostic composition of the group members on participants’ ratings of friendly (*b* = 3.81, SE = 1.78, *p* = 0.035), such that participants reported greater friendliness when more members of the group were autistic. Finally, there was a significant actor effect of diagnosis on ratings of awkwardness (*b* = 10.62, SE = 3.30, *p* = 0.002), with non-autistic participants reporting less awkwardness than autistic participants, and a significant partner effect of diagnosis (*b* = −9.02, SE = 4.17, *p* *=* 0.033), with participants reporting significantly less awkwardness when their group members were autistic compared to when their group members were non-autistic.

**Table 3.** Multilevel modeling estimated mean rapport ratings as a function of participant diagnosis and group composition.

Rapport item	Diagnostic composition of other group members		
	All same diagnosis	Majority same diagnosis	Majority different diagnosis
Easy			
Non-autistic	85.4	85.0	84.1
Autistic	87.8	81.0	67.4
Enjoy			
Non-autistic	85.4	83.0	78.2
Autistic	83.3	77.9	66.9
Friendly			
Non-autistic	88.6	89.2	90.4
Autistic	91.0	86.6	77.6
Success			
Non-autistic	88.2	87.5	85.9
Autistic	88.9	86.3	81.1
Awkward			
Non-autistic	67.9	72.7	82.4
Autistic	64.7	57.5	43.1
Composite			
Non-autistic	414.7	417.8	424.0
Autistic	415.2	389.7	338.8

To explore why rapport may have been higher in same-diagnosis groups relative to mixed groups, we pursued several post hoc observations quantifying behaviors on the Jenga task that may indicate differences in collaboration and problem-solving strategies between the four group types. We counted the number of all-autistic, all-non-autistic, and mixed groups that included a group member who built a separate Jenga tower on their own, had at least one participant not participate, and/or had participants begin one tower before restarting and building another. Having a group member build a tower on their own or not participate at all may indicate less collaboration and inclusion in the group activity, while instances of rebuilding a tower may indicate differences in planning and problem-solving relative to groups who pre-plan and build the tower only once. As can be seen in Table 4, none of all-autistic groups demonstrated any of these three behaviors, whereas seven instances occurred in the non-autistic groups, and nine in the mixed groups.

**Table 4.** Collaborative number of all-autistic, all-non-autistic, and mixed groups in which at least one participant rebuilt a tower, built a separate tower, or did not participate.

Group type	Rebuilt tower	Participant built a separate tower	Not all members participated
All-autistic (*n* = 13)	0	0	0
All-non-autistic (*n* = 10)	3	4	0
Mixed (*n* = 13)	1	6	2

## Discussion

Although prior research has demonstrated that autistic adults often develop better rapport with autistic compared to non-autistic partners during dyadic interactions (Crompton, Sharp, et al., 2020; Morrison et al., 2020; Rifai et al., 2022), no previous study has experimentally examined rapport among and between autistic and non-autistic people in group settings. In this study, we systematically manipulated the diagnostic composition of four-person groups to determine whether autistic and non-autistic people vary in their feelings of rapport depending on the composition of their group in terms of autism status. In contrast to a social deficit model of autism, autistic adults reported high rapport when in groups with other autistic adults. In fact, the all-autistic groups reported significantly higher levels of enjoyment and friendliness than the mixed groups and generally provided the highest rapport scores of any group type. It may be the case that group composition more directly affected rapport ratings of enjoyment and friendliness than ratings of “easy” and “successful” because the former may relate more to perceptions of interpersonal positivity whereas the latter may be more dependent on task success. Regardless, these findings align with the DEP (Milton, 2012) and other recent studies demonstrating that autistic adults often experience enhanced social experiences with one another (Crompton, Hallett, et al., 2020; Crompton, Sharp, et al., 2020; Crompton, Ropar, et al., 2020; Crompton et al., 2023; Morrison et al., 2020; Rifai et al., 2022), extending these results to group contexts.

Consistent with our hypotheses, rapport for autistic adults was the highest in the company of other autistic participants and declined when in the company of non-autistic group members. Differences in thinking, communication, and collaborative preferences between autistic and non-autistic participants may have negatively affected rapport ratings for autistic participants in the mixed groups. Autistic people can struggle to connect with non-autistic people and often adopt conscious and unconscious masking strategies to fit in non-autistic spaces (Pearson & Rose, 2021). Masking requires significant cognitive resources (Hull et al., 2017) that may have been particularly taxed in group settings given the heightened group demands and potential need to interact with several non-autistic partners at once. In addition, because participants were informed of the diagnostic composition of their group prior to beginning the interaction, autistic participants may have felt reduced pressure to mask in all-autistic groups but increased pressure in mixed groups, affecting their feelings of rapport and connection with group members. Critically, findings were based on self-report of their personal experience within the groups, and it is unclear how rapport manifested observationally. Future studies are encouraged to examine whether the basis for autistic rapport may differ linguistically and behaviorally from non-autistic rapport; it may be that traditional indicators of rapport—reciprocity, synchrony, eye contact, affect—may not extend to autistic interactions.

In contrast, and contrary to our hypotheses, rapport was relatively consistent for non-autistic adults across group types. Unlike autistic adults, they reported similar levels of rapport when interacting with both non-autistic or autistic adults, or when in the neuromajority or neurominority. We had predicted that non-autistic people would be less comfortable and more misunderstood in autistic company, leading to reductions in connection and rapport. However, only autistic people expressed reduced rapport when in the minority, suggesting that autistic people may be more sensitive than non-autistic people to the diagnostic composition of the group. Greater sensitivity of autistic people to their social context would be logical in the context of evidence that autistic people commonly have a history of negative social experiences (Acker et al., 2018; Goddard & Cook, 2022; Grace et al., 2022). In contrast, non-autistic people might have felt relaxed and confident based on a wealth of past experience, even when put into a situation where they were in a minority.

There are also several other possible reasons why group composition affected rapport to a greater degree for autistic compared to non-autistic participants. First, the diagnostic composition of each group was disclosed to participants. Diagnostic disclosure improves non-autistic impressions of autistic adults (Sasson and Morrison, 2019) but does not affect impressions formed by other autistic people (DeBrabander et al., 2019). Furthermore, non-autistic people also perceive themselves to be more helpful to autistic people when informed of their diagnosis (Heasman & Gillespie, 2019). It is possible, therefore, that non-autistic adults in this study were differentially affected by knowing when they were interacting with autistic people and increased their rapport ratings to be more inclusive. This may be particularly relevant in this sample due to the characteristics of participants, who were mostly in their twenties and thirties; many were college students, who tend to hold more progressive views of neurodiversity relative to the general population (Weakliem, 2002).

Autistic and non-autistic people also can differ in problem-solving strategies (Best et al., 2015; Paola et al., 2021), and they may have engaged in distinct collaborative behaviors on the Jenga task that differentially affected rapport. Social identity can influence team collaboration and creativity by shaping how diverse perspectives are integrated (Gino et al., 2009). This integration is often challenging due to biases and stereotypes based on identity factors like gender or ethnicity (Van Knippenberg et al., 2003). Teams with mixed identities often dismiss others’ views (Matlz & Kohli, 1996), hindering effective information integration (Jaworski & Kohli, 1993; Slater & Narver, 1995). Exploratory observations indicated that, in contrast to the other groups, every member of the all-autistic groups participated in the tasks; no participants in the all-autistic groups built a separate tower from the rest of the group, and no all-autistic group ever rebuilt a tower after already beginning one. These behaviors were all present in the non-autistic groups and in mixed groups and occurred most frequently in the mixed groups. Although speculative, instances of working alone and not participating may be indicative of disagreement or dissatisfaction between group members, while instances of rebuilding towers may indicate different planning and problem-solving strategies than used by the all-autistic groups. Future studies are encouraged to examine objective measures of collaborative success (e.g. tower height and collaborative behaviors) in group settings involving both autistic and non-autistic individuals.

Some limitations should be kept in mind when interpreting the findings from this study. First, although the overall sample size of autistic and non-autistic participants was sufficient, there were only 13 neurominority groups in total, reducing the power to detect some group differences, and interactions between diagnosis and minority status, and potential moderating effects of other participant characteristics. This low power also affected how analyses were pursued, with effects for individual rapport items uncorrected for multiple comparisons. Thus, item-level results should be considered preliminary and require corroboration through replication. The sample size also precluded including an additional group condition with an equal mix of autistic and non-autistic participants. Future research could examine whether autistic and non-autistic participants in such a context would be more likely to interact and build rapport with those who share their diagnostic status, as has been seen outside experimental settings (Chen et al., 2021). Furthermore, we cannot rule out that some participants may have had prior familiarity with one another. Although we recruited from many locations and sources to reduce the chances that this occurred, and we have no reason to expect that familiarity would differ systematically across the four group types, future studies should correct for this limitation.

Shared demographic variables, including race, gender, and age, may contribute to positive social interactions, as people tend to identify more readily with in-groups on these characteristics (Hewstone et al., 2002). Our modest sample size meant we were unable to ask whether other differences in participant characteristics affected rapport ratings, including differences between self-identified and formally diagnosed autistic participants. Minority members of a group, such as gender or racial minorities, often experience greater cognitive preoccupation with their group membership and report lower levels of positive affect in group settings compared to majority members (Lüken & Simon, 2005). Future research should attempt well-powered studies to measure the independent and intersectional effects of multiple minority statuses on autistic and non-autistic rapport. The sample also lacked diversity in several ways that may limit generalizability. No participants had intellectual disabilities, none were non-speaking, and the majority were White, in their twenties, and identified as female. Although comparable on most demographic characteristics, the autistic and non-autistic participants differed on gender composition. In particular, more autistic participants self-identified as non-binary, which is consistent with other studies (Øien et al., 2018; van der Miesen et al., 2018), although it is also not evident that this group difference would be able to explain the patterns of results reported here. In addition, included autistic participants were also comfortable being part of a study in which the diagnostic composition of the groups was disclosed, which may have resulted in a selection bias. Future research could examine whether rapport in mixed groups differs when diagnoses are disclosed or undisclosed. Pre-existing expectations and biases, such as attitudes toward interacting with autistic or non-autistic people, as well as the stress and anxiety associated with a desire to belong, may vary depending on whether diagnoses are known. Finally, we did not measure masking behaviors. Many autistic people consciously and unconsciously mask their autistic characteristics to fit in and avoid discrimination (Pearson & Rose, 2021), and this may have occurred more in the presence of non-autistic group members. Rapport may have differed depending on the presence, degree, and effectiveness of masking behaviors.

Despite these limitations, this study furthers our understanding of interactions among autistic and non-autistic adults by extending investigations to group contexts and offers information on how composition affects rapport differently for autistic and non-autistic people. Autistic participants benefited from interacting with group members that shared an autism diagnosis and were more sensitive to group composition compared to non-autistic participants, who displayed more consistent rapport ratings regardless of group composition. Overall, these findings largely align with the DEP in group contexts and may serve as the foundation for future research. This information may also be explored in applied settings where group work is common (e.g. education) to promote more inclusive environments for autistic people.
